# Crop Landraces and Indigenous Varieties: A Valuable Source of Genes for Plant Breeding

**DOI:** 10.3390/plants13060758

**Published:** 2024-03-07

**Authors:** Efstathia Lazaridi, Aliki Kapazoglou, Maria Gerakari, Konstantina Kleftogianni, Kondylia Passa, Efi Sarri, Vasileios Papasotiropoulos, Eleni Tani, Penelope J. Bebeli

**Affiliations:** 1 Laboratory of Plant Breeding and Biometry, Department of Crop Science, Agricultural University of Athens, Iera Odos 75, 11855 Athens, Greece; e.lazaridi@aua.gr (E.L.); mgerakari@aua.gr (M.G.); stud117006@aua.gr (K.K.); sarri@aua.gr (E.S.); vpapasot@aua.gr (V.P.); etani@aua.gr (E.T.); 2 Institute of Olive Tree, Subtropical Crops and Viticulture (IOSV), Department of Vitis, Hellenic Agricultural Organization-Dimitra (ELGO-Dimitra), Sofokli Venizelou 1, Lykovrysi, 14123 Athens, Greece; kapazoglou@elgo.gr; 3 Department of Agriculture, University of Patras, Nea Ktiria, 30200 Messolonghi, Greece; k.passa@upatras.gr

**Keywords:** alfalfa, cowpea, genome wide association studies, genomic tools, genomics, local populations, marker assisted selection, melon, tomato, woody perennial crops

## Abstract

Landraces and indigenous varieties comprise valuable sources of crop species diversity. Their utilization in plant breeding may lead to increased yield and enhanced quality traits, as well as resilience to various abiotic and biotic stresses. Recently, new approaches based on the rapid advancement of genomic technologies such as deciphering of pangenomes, multi-omics tools, marker-assisted selection (MAS), genome-wide association studies (GWAS), and CRISPR/Cas9 gene editing greatly facilitated the exploitation of landraces in modern plant breeding. In this paper, we present a comprehensive overview of the implementation of new genomic technologies and highlight their importance in pinpointing the genetic basis of desirable traits in landraces and indigenous varieties of annual, perennial herbaceous, and woody crop species cultivated in the Mediterranean region. The need for further employment of advanced -omic technologies to unravel the full potential of landraces and indigenous varieties underutilized genetic diversity is also indicated. Ultimately, the large amount of genomic data emerging from the investigation of landraces and indigenous varieties reveals their potential as a source of valuable genes and traits for breeding. The role of landraces and indigenous varieties in mitigating the ongoing risks posed by climate change in agriculture and food security is also highlighted.

## 1. Introduction

Landraces and indigenous varieties constitute an important reservoir of genetic diversity [1], conferring tolerance to abiotic and biotic stresses, and providing superior nutritional and sensory quality products as well as stability of performance in adverse environments [2,3,4]. Landraces (primitive varieties, farmers’ varieties, traditional varieties, local varieties, folk varieties) are dynamic populations that have a historical origin, distinct identity, and lack formal crop improvement, being also genetically diverse, locally adapted, and associated with traditional farming systems [5]. In general, the term ’landrace’ encompasses a plethora of concepts that have been diversified through time [2].

Historically, the cultivation of landraces and indigenous varieties enabled farmers to cope with harsh weather conditions and several biotic stressors. Hence, landraces played a pivotal role in ensuring food security mainly due to their rich genetic diversity [6]. The use of landraces remained unaltered until the rise of formal plant breeding which led to the development of high-yielding varieties that gradually replaced landraces and indigenous varieties [5,7]. Nowadays, landraces and indigenous varieties, although they remain in most cases a neglected and underutilized material, gain attention once more due to the increase of genetic erosion (the decline in crop diversity due to monoculture of modern crop cultivars that are genetically homogeneous) [8,9] and the growing demand for more diverse and local food products by the consumers [4].

As climate changes drastically, global food security is seriously threatened [10]. Many crop landraces and indigenous varieties, of cowpea, melon, tomato, apple, and grape, are cultivated and maintained in situ in Southern European countries, such as Greece, Italy, Portugal, and Spain [11,12]. The phenotypic and genetic diversity of landraces and indigenous varieties has been assessed thoroughly for many species around the Mediterranean [13,14,15,16,17,18,19,20]. These studies revealed the adaptive capacity of this material to novel conditions and its potential use in breeding programs. Therefore, landraces and indigenous varieties germplasm constitutes a valuable source for breeding resilient cultivars, focusing on adaptation traits under the rapidly changing climatic conditions [21].

Using DNA markers that are linked to targeted characteristics, molecular breeding facilitates the selection of genotypes with desirable traits. Quantitative trait loci (QTLs) related to abiotic and biotic stress resilience, high yield, quality traits, and high nutritional value have been identified in several landraces [22,23,24,25]. Marker-assisted selection (MAS) including marker-assisted backcrossing (MABC), gene pyramiding, marker-assisted recurrent selection (MARS), and genomic selection (GS), aid in the identification of alleles and the transfer of desired genes [26].

During the last two decades, genome-wide association studies (GWAS) have greatly contributed to unraveling the genetic diversity of many landraces [27,28,29,30]. GWAS facilitates the rapid and accurate identification of alleles linked to preferable traits [31,32,33,34]. Compared to bi-parental populations, the GWAS panels have greater rates of recombination, which leads to higher mapping resolutions [35]. Furthermore, the ability to search for superior alleles and uncover genotype-phenotype associations among unrelated individuals is provided by association mapping research’s exploitation of broad genetic heterogeneity [36]. For GWAS, the selection of an appropriate working population and a plethora of markers for germplasm genotyping is therefore crucial. Thus, single nucleotide polymorphisms (SNPs) are the markers of choice, due to the availability of rapid and affordable genotyping assays. In addition, the development of new genomic tools and technologies, such as next-generation DNA/RNA sequencing and gene editing (e.g., CRISPR/Cas9), which can facilitate the identification, functional characterization, and/or alter the expression of genes of interest, can significantly affect climate change adaptation of commercial varieties through the transfer of favorable genes from landrace germplasm [37,38].

With the assistance of new genomic technologies and tools, landraces of crop species with desirable agronomical and quality traits have been exploited and the genes controlling these traits have been introgressed into the background of elite germplasm through conventional breeding methods [39,40,41,42,43,44,45,46,47]. Favorable genes related to abiotic and biotic stress tolerance have also been introgressed in farmers’ preferable landraces [48,49,50].

This review focuses on the importance of landraces and indigenous varieties as valuable genetic sources for breeding and gives a comprehensive overview of the progress in new genomic tools and their implication in crop improvement by identifying desirable traits from landraces and indigenous genetic material. We focused on annual and perennial crop species as well as indigenous varieties of woody perennial crops cultivated around the Mediterranean region. Cowpea, tomato, and melon were selected as representatives of annual Mediterranean crops due to the large number of landraces that are cultivated in the area, whereas alfalfa, as well as woody perennials; grape, and apple, are presented due to their high economic importance.

## 2. Application of New Genomic Technologies in Landraces and Indigenous Varieties of Cultivated Crops

### 2.1. Cowpea

Cowpea (*Vigna unguiculata* (L.) Walp.) is a diploid (2n = 2x = 22) warm season legume with landraces being the most common cultivated genetic material (conserved on-farm) [51]. A greater number of cowpea landraces (22,161) than the number of advanced/improved cultivars (516) and breeders’ lines (771) is also conserved ex situ in genebanks worldwide [52]. The implementation of multi-omics tools in cowpea breeding, including genomics, transcriptomics, proteomics, metabolomics, and inter-woven technologies [53], holds great promise for harnessing this diversity, enabling gene transfer from landraces to improved/modern/elite cultivars and facilitating the identification of loci and candidate genes, ultimately leading to accelerated genotypes’ selection and development of new superior varieties.

Advances in QTL mapping of cowpea traits resulted in the identification of numerous QTLs [51,54] that led to significant progress in genome sequencing. For QTL analysis, recombinant inbred lines (RILs) have been used; some of wich were derived from crosses between domesticated and wild cowpea [55,56,57], between cowpea and yard-long bean [58,59,60], or between wild cowpea and yard-long bean [23,61].

Landraces have also been used as parents for RILs’ development, especially regarding resistance to various biotic stresses and related genetic loci [62,63]. Landraces that served as parents to form RIL or multi-parent advanced generation inter-cross (MAGIC) populations with resistance to pests and diseases are presented in Table 1. A landrace with a speckled black and purple seed coat and purple pod tips ‘Sanzi’ was also used as a parent for a RIL in a study of identifying genes related to seed coat and pod tip color [64]. Furthermore, one of the eight founder parents of a MAGIC population, created to identify easily measurable traits associated with high above-ground biomass, was a landrace named ‘Yacine’ that is characterized by high phosphorus efficiency [65].

New breeding technologies for legumes have taken longer to develop than those for cereal crops [70]. Nevertheless, nowadays, the development of next-generation sequencing (NGS) technologies and platforms provides large-scale, genome-wide marker information which enables targeting important traits and breeding for climate-resilient varieties. GWAS also offers the ability to screen rapidly a plethora of interesting phenotypes, analyze their full genome, and identify loci for desirable traits. Therefore, they are valuable tools for screening landrace genetic material that harness useful genes associated with phenological traits, morphological traits, abiotic and biotic stress resilience, and high nutritional value.

Using the 51,128 SNPs, Cowpea iSelect Consortium Array, Muñoz-Amatriaín et al. [71] applied high-density genotyping, to assess the genetic diversity of a diverse cowpea col-lection including landraces. In this study, loci linked to agronomic and physiological traits, such as flowering time, were identified through GWAS. Paudel et al. [72] used 368 cowpea accessions (including landraces) and conducted GWAS managing to identify seven candidate genes associated with flowering time. Additionally, Seo et al. [73] performing GWAS on a collection of 384 Korean cowpea genotypes including landraces, identified four major candidate genes significantly associated with flowering time. Many other GWAS studies in collections that included landraces’ material, revealed candidate genes for phenological and morphological traits in cowpeas and other *Vigna* species [72,73,74,75,76,77,78].

So far, several cowpea landraces that possess genes involved in abiotic stress tolerance and biotic stress resistance have been utilized in conventional breeding schemes [51,79]. In molecular breeding, GWAS has proven to be a valuable technique in associating genomic regions with interesting traits such as drought and heat tolerance, mainly using breeding lines, MAGIC populations of cowpea, and to a very small extent landraces material [80,81,82]. MARS has been applied in research investigations screening for drought tolerance [83]. Molecular assisted backcrossing (MABC) was also used to introduce QTLs for drought tolerance as well as other QTLs related to pest and weed resistance from two IITA breeding lines to a cowpea landrace ‘Moussa Local’ [84].

Various plant morphological and physiological traits related to drought tolerance have been used in cowpeas as well. However, the QTLs discovered provide information only for tolerance occurring during a specific plant growing stage [85,86]. A GWAS was therefore conducted to associate drought tolerance with two stomata-related traits, namely critical soil water content and slope of transpiration rate declining, using 113 cowpea accessions, mainly landraces, and a set of 434 SNPs [86]. Thirty-nine SNP loci were found to be associated with drought resistance of yard-long bean (*Vigna unguiculata* ssp. *sesquipedalis*) via GWAS of a RILs collection that was derived from a cross which landrace ‘ZN016′ was one of the parents [87,88].

Unlike other major grain legumes (e.g., soybean, chickpea), limited progress has been achieved toward the identification of QTL(s) affecting salinity tolerance in cowpeas [89]. Association of SNPs with salinity tolerance of a cowpea collection, including landraces, was identified by Ravelombola et al. [90] using genotyping-by-sequencing (GBS) analysis, in which three and seven SNPs were identified to be associated with tolerance in the germination and seedling developmental stages, respectively. More recently, a total of 234 MAGIC populations along with their 8 founders were evaluated for salt tolerance under greenhouse conditions, using 32,047 filtered SNPs [91].

Identification of genes associated with cowpea biotic stresses has been assessed through GWAS using, among others, genetic material from landraces (Table 2). GWAS was performed to identify loci associated with crops’ nutrient status, enhancing biofortification efforts. In one of these studies, seven loci were identified through GWAS that were associated with the carotenoid content of sprouts of 125 cowpea accessions including landraces [92]. Moreover, a GWAS was undertaken recently on 161 accessions, including landrace material to identify markers related to cowpea seed protein content, in which three candidate genes were identified [93].

Despite the low transformation efficiency rates observed in cowpea, genome editing tools, like CRISPR/Cas9, have begun to be implemented in cowpea breeding (using *A. tumefaciens* and *A. rhizogenes* as carriers) with encouraging results [98,99,100,101,102,103,104]. Thus far, the plant material used for CRISPR/Cas9 consisted of cowpea varieties and breeding lines. Nevertheless, improving transformation efficiencies and regeneration rates of other genotypes will render this technique a powerful and promising tool for employing gene editing in landrace genetic material as well.

### 2.2. Alfalfa

Alfalfa (*Medicago sativa* L.) (2n = 4x = 32) is a major legume crop, essential to the sustainability of agriculture [105,106]. Because of its high biomass output, highly nutritious preserved fodder (hay and silage), and extensive environmental adaptability, it is considered one of the major forage legumes worldwide. Restricted genetic variability of crops may result in crop losses due to pests as well as inadequate nutritional value of products. Furthermore, to feed the world’s estimated population of 9.7 billion people by 2050, agriculture production [107], including legumes, must be raised by 70% [108,109,110,111].

Agronomic and quality traits of alfalfa can be improved to enhance overall productivity by using germplasm with high genetic variation, i.e., landraces. There are 3367 *Medicago sativa* accessions conserved ex situ in genebanks worldwide [52], while many alfalfa landraces are maintained in situ across different countries and biogeographical regions of Europe [11] that could be utilized in breeding programs.

Assessment of diversity levels, genetic structure, and distinctness of alfalfa cultivars and landraces is a prerequisite for breeding novel genotypes with desirable traits. Although the advancement of novel genomic technologies in alfalfa breeding is relatively recent compared to other crops, the application of genomic tools, such as molecular marker genotyping, sequencing, and genome assembly of alfalfa close relatives has significantly contributed to understanding genetic diversity, population structure, and gene expression patterns in landraces of *Medicago* species and has provided valuable insights into stress tolerance mechanisms [112,113].

The genetic diversity and population structure of a collection of tetraploid alfalfa (*Medicago sativa* ssp. *sativa* L.) including 156 landraces was assessed using 336 genotypes and 85 SSR markers [114]. The researchers identified a total of 1056 alleles, indicating high genetic diversity in tetraploid alfalfa germplasm. The analysis revealed that the highest genetic diversity exhibited in regions of chromosomes Chr2 and Chr3, and landraces showed greater diversity compared to wild materials and cultivars [114]. These findings provide valuable insights for genetic and genomic analysis, facilitating the effective utilization of genetic resources in alfalfa breeding. Another study explored the potential of GS for alfalfa yield improvement using GBS data [115]. Two genetically diverse reference populations, a landrace, and a modern variety, one adapted to a sub-continental climate and the other to a Mediterranean climate environment (Mediterranean population), were examined. The absence of sub-population genetic structure was noted in both populations. GWAS identified moderately associated SNPs, suggesting a lack of major-effect QTLs. GS outperformed conventional selection, with over three-fold greater than predicted yield gain per unit time, especially with shorter selection cycles.

The genetic diversity of 18 non-dormant alfalfa accessions (including ten landraces) was studied at the morpho-agronomic and molecular levels using sequence-related amplified polymorphism (SRAP) markers (loci), which are comparatively widely distributed across the plant genome [116,117]. The SRAP data distinguished widely dispersed alfalfa populations in various conditions and indicated suitable genetic resources for breeding [117]. SRAP markers were also used successfully to determine genetic diversity among Tunisian alfalfa genotypes, as well as to identify and rationally use local and foreign alfalfa populations for breeding programs, focusing on the development of new, high-yielding cultivars that are more efficiently adapted to North African water-deficit conditions [118].

Zhang et al. [119] compared genomic prediction (GP) methods, evaluating fall dormancy (FD) prediction accuracy by using a hybrid GWAS and GP model. They extended the GP model for predicting new traits using resequencing data and FD measurements from 220 accessions, including 95 landraces. The study investigated the importance of FD in alfalfa for winter resistance and cultivar selection. Methods like transcriptomics and QTL mapping identified key FD genes but lacked predictive accuracy. This research introduced machine learning, specifically what is known as Support Vector Machine (SVM) regression combined with GWAS management to achieve high levels of accuracy (64.1%). The findings underscore the potential of machine learning and GWAS markers for precise FD prediction, benefiting alfalfa genetic research and breeding.

Drought resistance is a critical breeding objective for increasing alfalfa productivity in dry and semi-arid environments. Therefore, the identification of drought tolerance-related genes will aid in breeding for improved drought tolerance and water use efficiency. A diversity panel of 198 alfalfa cultivars and landraces was used for the identification of loci associated with drought resistance traits using GWAS with GBS genotyping by Zhang et al. [120].

Jiang et al. [121] mapped leaf length, leaf width, and leaf area, traits that influence alfalfa forage yield and quality, in an F_1_ mapping population derived from a cross of a cultivar named ‘Zhongmu No.1’ that is characterized by large leaf area and a landrace named ‘Cangzhou’ which expresses a smaller leaf area. Using a combination of QTL mapping, RNA-seq analysis, and qRT-PCR, they identified seven candidate genes associated with leaf development in five major QTL regions [121]. Their study underlines the importance of landraces and provides a basis for marker-assisted breeding. Landrace ‘Cangzhou’ was also used as a source of early flowering paternal material towards the formation of two full-sib F_1_ populations attempting GS for biomass yield traits [122]. However, the prediction accuracy of GS depends on the number of markers used as well as on the population size, structure, and diversity [123].

The utilization of genomic tools in exploring the diversity and adaptive potential of landraces within the *Medicago* genus represents therefore a transformative leap in our understanding of these plant species. As we explore further the genomic landscapes of *Medicago* landraces, we uncover not just the genetic foundations of their distinctive traits but also the potential for improving agricultural sustainability and resilience. As the demand for sustainable and high-quality forage crops continues to rise, the application of genomic tools in alfalfa will play a crucial role in advancing the breeding of superior landraces, ultimately contributing to the sustainable management of agricultural systems. The majority of the studies including *Medicago* landraces that use new genomic tools have primarily remained in the exploration of genetic diversity, rather than introducing these technologies into breeding programs.

### 2.3. Tomato

Due to self-pollination, founder effects, natural and artificial selection, and excessive inbreeding of specific genotypes, *Solanum lycopersicum* (L.) (2n = 2x = 24) underwent many genetic bottlenecks during its domestication and evolution, particularly in Europe and North America [124]. The most successful domesticated tomato species are found in Mediterranean countries, particularly in Italy and Spain. These countries have been designated as secondary diversification centers for *S. lycopersicum* resulted in variation giving rise to a wide range of landraces grown for centuries and are still frequently found in local markets [125]. Tomato landraces serve therefore as a valuable source of genetic diversity, particularly for attributes like resistance to multiple environmental stressors and the production of high-quality fruit products. A large genetic pool consisting of landraces and wild relatives can be utilized in tomato breeding programs [126].

The application of next-generation sequencing (NGS) and high throughput genotyping can contribute extensively to the exploration and utilization of tomato landraces. Víquez-Zamora et al. [127] identified 6000 SNPs, 5528 of which were used to evaluate tomato germplasm at the species level, including landraces and tomato wild relatives’ accessions. Selecting a core collection of robust SNPs covering the whole tomato genome can be used for the development of future arrays [127]. In a similar study, patterns of polymorphism were examined, population structure was characterized, and putative loci were identified under positive selection through genotyping 214 tomato accessions, including cultivated landraces, commercial varieties, and wild relatives, via a custom-made Illumina SNP panel. The results revealed 175 successfully scored SNP loci which were found to be polymorphic [128].

Moreover, Carbonell et al. [129] investigated tomato variability of various landraces and traditional varieties from Spain and Italy. They applied backcrossing and MAS to simultaneously introduce three genes (*Tm-2a*, *Ty-1*, and *Sw-5*) from wild relatives to landraces, that confer resistance to pertinent viruses, like Tomato Spotted Wilt Virus (TSWV), Tomato Mosaic Virus (ToMV), and Tomato Yellow Curl Virus (TYLCV), using different types of molecular markers such as sequence-related amplified polymorphism (SRAP), amplified fragment length polymorphisms (AFLPs), simple sequence repeats (SSRs), SNPs, and transcription factor GATA4 probes. The goal of this research was to introduce resistance into traditional tomato varieties, without affecting their productivity and quality.

In a study conducted by Sacco et al. [130], 123 tomato landraces were collected from diverse geographical regions, intending to encompass a broad spectrum of diversity, applying GWAS. The genotyping process utilized the Solanaceae Coordinated Agricultural Project (SolCAP) tomato array platform, which analyzed a total number of 7720 SNPs. Across the entire collection, 87.1% of markers exhibited polymorphism, but this percentage was reduced to 44–54% when groups of genotypes with different origins were examined. These markers were also mapped onto tomato chromosomes, and 98 candidate genes were associated with fruit morphology traits assessed. The study identified six regions where candidate genes coincided with 19 linked SNPs. Additionally, 17 associated SNPs were in genomic regions lacking candidate genes. Based on the study of Sacco et al. [130], SolCAP data for 15 landraces were retrieved. Sites filtered for minor allele frequency (MAF) < 15%, resulted in 954 polymorphic SNPs, covering the whole tomato genome, that varied from 54 (Chr6 and Chr10) to 155 (Chr3) SNPs per chromosome; all genotypes also demonstrated decreased heterozygosity (H_o_) [131]. The discovery of such markers captured the high variability in the germplasm collection, while they could potentially serve as tools for selection in future breeding programs.

Moreover, two tomato landraces from Southern Italy were studied, named ‘Corbarino’ (COR) and ‘Lucariello’ (LUC). Both landraces have long shelf-life characteristics and high-quality fruit productivity under water deficit conditions in their traditional area of cultivation. Whole-genome resequencing of these two Italian landraces was conducted, using a newly developed pipeline named Reconstructor, revealing sequence variations in genes associated with traits such as drought stress tolerance, fruit quality, and long shelf-life. In the genome of both landraces specific regions were detected that exhibited similarity to those found in the wild species *Solanum pimpinellifolium* and *Solanum pennellii,* known for their reported high levels of drought tolerance. Small deletions and insertions of SNPs were also revealed [132]. Recently, RNA sequencing was applied to plants of COR and LUC landraces, while they were under drought stress conditions. A total number of 3089 and 2135 Differentially expressed genes (DEGs), including specific annotated genes, were identified for COR and LUC landraces, respectively. Regions of genes containing SNPs were revealed in the landraces’ genomes after comparison with the tomato reference genome. Enriched gene ontology (GO) categories also showed that genes affecting oxidoreductase activity, water use efficiency, nucleotide salvation, and lipid biosynthesis-related processes were enriched among up-regulated DEGs [133].

In another study, the genetic diversity of 64 tomato landraces from Campania, Sicily, and Apulia regions, with drought-tolerant and long self-life traits was assessed using an SNP markers array. A dendrogram, based on 1575 SNP loci, revealed four main clusters which were clearly differentiated. Structure analysis confirmed the presence of four genetic groups within the collection, with admixture between them. This study shows the potential for tomato landrace germplasm to be an innovative source of alleles with interest traits [134].

High-throughput genotyping of 10 tomato landraces revealed candidate genes related to high-temperature stress response [22]. The GBS approach was used for the exploration of the genetic variability of these landraces. Landrace ‘E42’ was found to be the most polymorphic, and the researchers supported that candidate genes/QTLs regulating heat tolerance in that specific landrace could be further studied to understand the genetic mechanisms that control traits relative to high and stable yields under heat stress [22].

Additionally, GWAS studies have also been widely used for screening populations with different favorable traits [135,136]. In a study conducted by Rodriguez et al. [29], 115 landraces were assessed, revealing that the use of RNA-Seq is effective not only for the identification of different genomic regions for traits such as fruit diversity but also for traits related to plant adaptation to climatic change. Moreover, different marker-trait associations were mapped on chromosomal regions, validating results for previously reported candidate genes, related to the same traits [29].

It is important to highlight the role of tomato landraces for their unique adaptability under various environmental stressors along with their high-quality features, as they comprise a powerful tool in breeders’ hands to deal with the changing climatic conditions worldwide and produce more sustainable products, as well as to meet the demands of consumers for healthier and safer food resources [137]. The application of current genomic tools and technologies such as MAS, NGS, and GWAS can contribute effectively to the development of faster and sustainable new tomato breeding strategies, creating commercial well adapted to the changing environmental conditions varieties through landraces germplasm utilization [16,129,133].

### 2.4. Melon

Melon (*Cucumis melo* L., 2n = 2x = 24) is an annual herbaceous plant that belongs to the family of *Cucurbitaceae* [138]. With a genome size of 450 Mbp, *Cucumis melo* L. is perhaps the most genetically diverse crop among the *Cucurbitaceae* [139,140]. Melon is cultivated worldwide; however, it mainly grows in temperate, subtropical, and tropical regions. For hundreds of years, melon landraces have been widely cultivated in marginal regions due to their rich genetic variation and their capacity to cope with adverse environmental conditions. For this reason, this material is considered a valuable repository of beneficial genes to be exploited in crop breeding. Nowadays, the amount of genetic and genomic data has increased significantly, while a wide range of genomic tools are available for exploring and harnessing landraces’ genetic diversity, fostering the identification of genes responsible for desirable traits [141].

Recently, molecular markers (i.e., SSRs, SNPs, etc.) have been utilized in melon landraces, for the study of genetic diversity, molecular fingerprinting of genetic material, and association with variable traits such as resistance to biotic and abiotic stresses. Forty-nine Indian landraces and varieties were studied using SSR markers, revealing beneficial alleles at loci conferring resistance to Fusarium wilt, downy mildew, powdery mildew, aphids, and viruses [142]. Using GBS sequencing, Pavan et al. [143] detected 25,422 SNPs by examining 72 accessions of Apulia in Southern Italy, including landraces of a winter melon, namely chate melon, which is also known by the folk name of ‘Carosello’ and ‘Barattiere’ that was never characterized before. Subsequently, by carrying out GWAS they identified two SNP loci associated with flowering time of male flowers and two others associated with seed width. In a similar study by Gur et al. [144], using GBS in a collection of 177 accessions including landraces, 23,931 SNPs from a total of 99,263 SNPs were selected through filtration for GWAS analysis. Fruit shape, flesh color, and sex determination were effectively mapped to short genomic intervals with the most important SNPs discovered for each trait located within less than 100 Kb from previously mapped causative genes.

GBS and GWAS were also used by Wang et al. [145] on 2083 melon accessions of the U.S. National Plant Germplasm System. Their results gave preliminary information for the evolution and dissemination of melon in various geographic locations and provided a plethora of SNPs in known QTL regions underlying fruit quality and other horticultural traits, namely sex expression, ovary pubescence, leaf and fruit morphology, and flesh soluble solid content [145]. Moreover, GBS was applied on 47 accessions belonging to Spanish melon landraces to perform an in-depth study and characterization of this melon germplasm collection [146]. To examine the genetic diversity of prevailing varieties in China, Target-seq technology was utilized by Zhang et al. [147] to screen out genome-wide SSRs and SNPs from 149 re-sequenced accessions and established the DNA fingerprint of 259 accessions of which 84 were landraces. Two distinct SNP loci were revealed for the identification of the ssp. *agrestis* varieties, while a core set of 40 SNPs and 23 SSRs was chosen to identify, discriminate, and protect intellectual property rights. Moreover, the increase in gene exchange between ssp. *melo* and ssp. *agrestis* could potentially improve the genetic diversity of the ssp. *agrestis* with important implications in breeding.

A whole genome resequencing of 91 melon landraces from India and East Asia (297 accessions including wild types and improved cultivars) revealed selective sweeps and new GWAS signals linked to fruit size, flesh thickness, and aroma accumulation and revealed a candidate gene (*CmCLV3*) for melon carpel number variation [148]. Lian et al. [149] developed melon mapping populations by crossing ‘SD119’, a Chinese landrace (conomon group) and ‘HG118’, a Chinese inbred line. They reported fruit size QTL analyses and high-resolution genetic maps improving our understanding of the genetic underpinnings of melon domestication and differentiation. Furthermore, two loci related to fruit size were identified on Chr5 and Chr11 and an auxin response factor and a YABBY transcription factor were suggested as the underlying genes.

Melon landraces also play a significant role as resistant plant material sources against several viruses. Tamang et al. [150] detected two QTLs in Chr5 and Chr3 associated with Cucurbit Yellow Stunting Disorder Virus (CYSDV) resistance using as plant material an F_2:3_ population derived from crossing between PI 313970, an Indian landrace resistant to CYSDV, and ‘Top Mark’, a cantaloupe melon susceptible to CYSDV.

RNA-sequencing was conducted to identify DEGs associated with Tomato Leaf Curl New Delhi Virus (ToLCNDV) between an Indian resistant melon landrace ‘WM-7’ and a Spanish susceptible cultivar ‘Piñonet Piel de Sapo’ [151]. They suggested that a gene in Chr11 encoding a Glutaredoxin protein is the most promising candidate for resistance to ToLCNDV. Furthermore, they identified several SNPs on structural functionality of DEGs, associated with several genomic regions (Chr2, Chr11, Chr12), having also different types of impact, e.g., moderate (a missense change causing an amino acid alteration) and high (introduction of a stop codon), thus significantly influencing ToLCNDV resistance.

Transcriptomic analysis by Ling et al. [152] revealed 293 genes upregulated in the resistant to downy mildew (DM) cultivar PI 442177 in comparison to the susceptible landrace ‘Huangtu’, while significant enrichment was also observed in pathways associated with defense response in PI 442177. Moreover, two landraces, ‘Huangtu’ and ‘Huangdanzi’, susceptible to DM were used with PI 442177, to produce F_2_ populations for linkage analysis, resulting in the identification of a major QTL, *DM9.1*, for DM resistance on Chr9 [153]. Landraces from different geographic regions were used by Oumouloud et al. [154] to develop functional markers that would improve MAS for the melon Fusarium wilt resistance gene (*Fom-2*).

Although the draft genome of melon was released in 2012 [155], the first high-quality genome assembly was generated by Zhang et al. [156], using the melon ‘Payzawat’. In their study, whole genome resequencing using single-molecule real-time (SMRT) sequencing technology of 50 Chinese melon accessions including landraces enabled them to identify and validate candidate mutations for genes controlling desirable traits, e.g., flowering, fruit morphology, ripening behavior, sugar content, etc.

Oren et al. [157] constructed the first pan-genome in melon by sequencing and de novo assembling 25 diverse melon inbred accessions revealing significant variation in genome size and structure among them. Recently, a more accurate genome assembly of ‘Mapao’ melon landrace was produced by Lyu et al. [158] by HiFi long reads and High-throughput chromosome conformation capture (Hi-C) technologies. Moreover, they constructed a pan-genome atlas and conducted GWAS to screen SNPs/InDels/SVs associated with agronomic traits related to the sweetness and appearance of melon fruit.

DNA microarrays have been used for transcriptome profiling of melon landraces. Gene expression analysis of Watermelon Mosaic Virus (WMV) infected melon plants was studied on a dataset of 17,443 unigenes represented on the melon microarray by employing the Spanish landrace ‘Tendral’ as the susceptible control [159]. Moreover, Saladié et al. [160] conducted a transcriptomic analysis of melon fruit ripening behavior with samples taken from a population developed from a cross between an inbred line of Spanish cultivar ‘Piel de Sapo’ and the Korean landrace ‘Songwhan Charmi’ (PI 161375), suggesting that classification of melon fruit ripening behavior into only two discrete categories is not accurate. Finally, Nieto et al. [161] conducted Eco-TILLING in 135 *C. melo* landraces and traditional cultivars among other accessions of melon wild relatives to identify new alleles of *eIF4E* that control resistance to Melon Necrotic Spot Virus (MNSV).

With the advent of modern genomic technologies, there is a great amount of new genomics data and innovative tools available for deciphering the genetic basis of complex traits in melon [162,163]. Melon landraces have the potential to be a significant gene pool to increase genetic variability and to introduce new beneficial traits particularly related to climate change into modern melon cultivars, providing thus significant prospects in melon breeding programs [15].

### 2.5. Grapevine

Grapevine (*Vitis vinifera* L.) (2n = 38) is one of the most economically important woody perennial crops cultivated around the world. Total vineyard acreage amounts to approximately 7.2 million hectares (mha) with annual grape production of ~77.8 million tons of which 47.4%, 44.5%, and 8% correspond to wine, table, and dried grape, respectively [164]. Grapevine domestication dates to antiquity (8000–11,000 years ago) and the *Vitis* genus encompasses 60–80 species (*Vitis* spp.) with purportedly very high genetic diversity. Based on a combination of ampelographic and genotyping studies approximately 6000–10,000 *Vitis vinifera* cultivars have been estimated to exist worldwide [165,166,167]. Nevertheless, only a small fraction of this wide genetic pool has been exploited for commercial use whereas the largest part of the diverse grapevine germplasm remains underexplored [168].

Over the last decades, a wealth of reports has been published concerning the genotyping of the indigenous grapevine reserves in the Mediterranean, either through SSR or SNP methodology [169,170,171,172,173,174,175,176,177]. In view of the drastic climate change and its impact on grapevine yield and quality, efforts have been undertaken to capitalize on the rapid advancements in genomic technologies to face current and future environmental challenges. These efforts aim to uncover significant associations between the rich grapevine diversity of indigenous varieties and a wide range of valuable agronomic traits at the molecular level [168,178]. In addition, grapevine performance relies intensely on appropriate rootstock utilization. Traditionally, European grapevine has been grafted onto interspecific North American hybrid rootstocks to combat phylloxera (*Daktulosphaira vitifoliae*) infestations and avoid its devastating consequences [179,180]. Cultivated grapevine varieties use a series of compatible rootstocks to secure productivity and disease resistance [181,182]. In total, 1343 rootstocks from 22 countries have been registered in the Vitis International Variety Catalogue (VIVC), however, most rootstocks used commercially originate from a few species, that is, *V. riparia*, *V. berlandieri*, *V. rupestris,* and their hybrids, thereby accommodating a very narrow genetic basis [171,173]. Choosing the appropriate rootstock ensures proper scion-rootstock compatibility and graft growth, consequently imparting the plant with the desired developmental, fruit quality, and stress resilience attributes [180,183,184,185]. The ongoing global climate change as well as future climate risk scenarios underscore the need for novel or improved rootstocks. Thus, the implementation of new genomic tools along with the exploitation of grapevine germplasm diversity is expected to address issues related to improved adaptability of both scions and rootstocks, contributing to sustainable and climate-resilient viticulture.

In recent years, a great number of targeted or GWAS studies have been reported aimed at dissecting the genetic basis underlying key traits related to grapevine quality and stress resilience [168,178]. One of the most important traits of the grape table and raisin industry is seedlessness. Seedlessness can arise from parthenocarpy (ovary growth without fertilization) or stenospermocarpy (early abortion of embryo development). The latter is a preferred breeding target as it produces appropriate-sized berries to meet the demands of the table grape market [186]. Early QTL mapping studies with biparental progenies revealed that a locus named *SdI* (Seed development Inhibitor) on Chr18 was related to variations in the spermostenocarpic trait and suggested *AGAMOUS 11* (a *MADS*-box gene involved in seed development) as the candidate underlying gene [186,187,188]. More recently, a GWAS conducted to investigate the genetic basis of seedlessness used an association panel of 199 genotypes (124 seeded/75 seedless) and 414,223 SNP markers. These genotypes were obtained from the National Grape Germplasm Repository at Zhengzhou Fruit Research Institute of the Chinese Academy of Agricultural Sciences and included indigenous varieties. The results suggested that a major genetic locus for the seedlessness trait may be located on Chr18 which is in agreement with earlier studies [189]. Further analysis detected 294 SNPs linked to the trait and proposed several genes known to be implicated in berry development and berry size as the underlying candidates. Interestingly, significant SNPs overlapped with an SSR marker (VMC7f2) which had been found associated with seedlessness in previous QTL studies [190].

In another GWAS investigation, a collection of 114 grapevine varieties (including indigenous varieties from Spain, France, Portugal, Italy) was used to explore the genetic basis of a successful fruit set, a major determinant of grapevine yield. Specifically, targeted sequencing of 289 candidate genes potentially involved in fruit sets across different varieties detected 164 SNPs in 39 genes associated with fruit set-related traits such as flower number, berry number, fruit set rate, coulure index, millerandage index, and seed number [191]. Interestingly, the SNPs identified were found to be more prevalent in genes encoding MADS-box transcription factors families implicated in the regulation of reproductive development processes and fruit formation such as *AGL6*, *AGL12*, *AGL21,* and *AP1*. These findings will aid in the elucidation of the molecular mechanisms underlying a complex trait such as fruit set with potential applications in grapevine yield optimization under climate pressures. Recently, a GWAS encompassing 588 cultivars (from a germplasm collection in Chile, including indigenous varieties and 536 segregating individuals from seven related F_1_ families from a Chilean breeding program) identified SNP markers significantly associated with pre- and post-harvest related traits. A candidate gene, *Vitvi11g000454* on Chr11, responsive to stress through jasmonic acid signaling, was linked to berry width with the potential for increasing berry size in grapevine breeding programs [192]. Furthermore, novel QTLs were discovered associated with post-harvest traits including decay, shriveling, and weight loss.

In a related abiotic stress project, a GWAS using 100 grapevine accessions (including interspecific hybrids used for fruit production, various rootstock varieties, and rootstocks selected in a breeding program) was undertaken to explore the genetic basis of stomatal conductance under drought stress and to identify genomic regions associated with drought tolerance mechanisms. SNP analysis detected 24 significant marker-trait associations and indicated 13 candidate genes as potentially responsive to water deficit [193]. Notably, one of these candidates encoded a raffinose synthase implying a role for this enzyme in the early response to drought stress cellular processes, in accordance with the known protective role of raffinose oligosaccharides against abiotic stresses [194]. Along these lines, a recent whole genome re-sequencing project was undertaken deploying 77 rootstock genotypes which encompassed Muscadinia and 12 other North American and Asian *Vitis* species and their hybrids to elucidate potential associations of stress-related traits with genomic regions. GWAS analysis identified six groups of 631, 13, 9, 2, 810, and 44 SNPs that were significantly linked to resilience to phylloxera, root-knot nematodes, salt, drought, cold, and waterlogging, respectively [195].

Similarly, a GWAS investigation exploring disease resistance mechanisms to the pathogenic fungus *Coniella diplodiella* causing white rot disease utilized 386 genotypes from Asian, North American, and European grapevine species, including indigenous varieties. Significant associations were detected between six SNPs located on Chr1, Chr2, Chr4, Chr13, Chr16, and Chr17 and the response to white rot disease whereas further analysis identified eight candidate genes linked to resistance [196]. These genes code for proteins related to defense mechanisms against biotic stresses and include receptor signal transduction kinases, pathogen effectors, a leucine-rich repeat receptor-like serine/threonine-protein kinase, an ethylene-responsive factor, and a zinc finger RNA-binding protein. Importantly, upon pathogen infection, these genes displayed marked upregulation in resistant *Vitis* accessions as compared to susceptible ones, indicating a functional role in successful response and resistance to white rot [197]. Identifying abiotic stress tolerance- and disease-resistant-associated genes in indigenous genotypes could be exploited in breeding programs aiming to confer resilience to commercial varieties or develop rootstocks with enhanced stress resilience properties [180,181].

In a recent study on the indigenous grapevine varieties from the Epirus region of Greece, an indigenous local variety named ‘Dichali’ was found to display tolerance to consecutive drought stress. Moreover, enhanced resilience to water deficit was accompanied by altered expression patterns of two microRNAs (miR157 and miR159) and genes encoding transcription factors (TFs) of the *MYB* and *TPR* families, suggesting epigenetic responses as well as the implication of potential miRNA/TF regulatory networks in dehydration tolerance [197]. A deeper understanding of the molecular mechanisms underlying key responses to environmental stressors would provide useful information for grapevine improvement and contribute to climate-smart viticulture.

Lastly, in the context of New Plant Breeding Technologies (NPBT) considerable efforts have been made via CRISPR targeted-editing tools to functionally characterize specific genes and unravel cause/effect relations for important traits, especially regarding disease resistance [198]. For example, a CRISPR/Cas9 system was employed for editing *VvMLO3* and *VvMLO4*, two genes associated with the defense response to mildew, leading to increased resistance to the fungus [199]. Similarly, editing a gene coding for the pathogenesis-related protein VvPR4b increased the susceptibility of grapevine mutants to downy mildew (*Plasmopara viticola*), indicating a role for this protein in the defense process [200]. CRISPR unquestionably constitutes a milestone in basic research for determining gene function and delineating molecular mechanisms. Nevertheless, significant advancement is required in grapevine transformation efficiency and regeneration capacity, particularly concerning valuable genotypes besides ‘Thomson Seedless’ and ‘Chardonnay’. In this regard, such progress is critical for exploiting the potential of this powerful tool and ensuring the success of targeted-genome-editing strategies at the application level.

### 2.6. Apple

Apple (*Malus domestica* Borkh.) (2n = 2x = 34) constitutes a major fruit crop grown worldwide, ranking third in economic importance after tomatoes and grapes with an annual production of about 95 million tons (12 million tons in Europe, 2022) [201]. It has been estimated that cultivated apples were domesticated around 3000 years ago and about 7500 apple varieties are cultivated worldwide [202]. Despite this high genetic diversity, only a limited number of apple genotypes are cultivated for commercial use. In fact, in the European Union, apple markets are dominated mainly by five elite apple varieties whereas the wide genetic pool of apple germplasm remains largely unexplored [202,203]. Ultimately, the few commercial varieties will tend to be increasingly vulnerable to climate change pressures reflected in compromised yields and quality.

Fruit quality such as apple aroma and taste as well as post-harvest properties are major concerns for the apple industry. Volatile organic compounds (VOCs) of fruits, phenolics, ripening traits, firmness, and texture constitute important targets in apple breeding. Abiotic stress tolerance and disease resistance, especially in the context of increased climate threats, are equally significant traits that have begun to be examined in apple research projects. Owing to a prolonged juvenile stage, self-incompatibility, and high heterozygosity, classical apple breeding is a laborious and time-consuming process [202]. Thus, efforts have focused on genomics-assisted breeding that would enable MAS for superior genotypes with enhanced quality and resilience at the seedling stage.

Over the past several years, investigations utilizing advanced genomic tools across the diverse apple germplasm have shed light on the genetic structure of numerous valuable agronomic traits [202,203,204]. For example, to contribute to genomics-assisted breeding, a GWAS with a collection of 172 apple accessions (including indigenous varieties) was performed [205]. The authors managed to link 55,000 SNPs with 10 phenotypic traits and revealed strong associations with loci for skin color, harvest date, and fruit firmness at harvest. Additionally, they detected significant associations for resistance to the devastating apple scab fungus (*Venturia inaequalis*). In relation to the latter, Švara et al. [206] have comprehensively reviewed current research on scab resistance and highlighted the need for integrating phenotypic, genetic, omics, and functional genomics approaches across the diverse apple germplasm in order to decipher molecular mechanisms underlying the anti-fungus defense process and achieve successful breeding for enhanced scab resistance [206].

Larsen et al. [207] associated SNP-marker data, generated through GBS experiments of an apple collection of 170 accessions, with fruit flavor, sugar, and acid content. This GWAS revealed strong marker-trait associations and identified candidate genes for aroma VOCs, sugar composition, and harvest date [208]. Subsequently, an extensive GWAS with over 1000 accessions from three different apple germplasm collections, including indigenous varieties, dissected further a previously identified large-effect locus associated with a NAC transcription factor gene (*NAC18.1*) linked to harvest date and firmness [208]. Their findings revealed additional polymorphisms in or around the gene encoding transcription factor *NAC18.1* that may lead to variation in these traits. Notably, the NAC-associated marker was indicated as a stronger predictor for firmness at harvest and at post-cold storage than three other markers used commonly by breeders based on genes that are involved in ethylene synthesis (*ACS1*, *ACO1*) and in pectin hydrolysis (*PG1*) [208,209]. Another large-scale GWAS exploring 21 fruit quality and phenology traits identified allelic variations in the *NAC18.1* gene which were associated with fruit-relevant traits including firmness features. In addition, other significant signals were detected on Chr15, Chr16, and Chr10 associated with phenolic content and fruit softening [210].

It should be mentioned that an essential tool for apple genomics-assisted breeding is the apple reference population (REFPOP), a collection of 534 genotypes composed of 269 apple accessions and 265 progenies from 27 parental combinations derived from recent European breeding programs [211]. A series of 30 traits related to phenology, productivity, tree vigor, and fruit quality were evaluated in the REFPOP collection during the period of three years and at six distinct locations in Europe with diverse climatic conditions (Belgium, France, Italy, Poland, Spain, and Switzerland). In addition, REFPOP was genotyped with high-density arrays (303K SNPs), thus providing the basis for GS and marker-assisted breeding across different environments. GWAS and GS studies utilizing REFPOP identified 59 stable-across locations and 277 location-specific associations, the majority of which were novel when compared to comprehensive publication datasets [212]. Integrating genetic data and phenotypic data assessed at diverse environments will reveal important trait–environment combinations and facilitate GS and prediction in apple breeding. Taken together, the employment of advanced genome-wide tools across the rich apple germplasm could be a promising strategy for detecting loci and defining genes associated with commercially relevant traits. Valuable insights arising from such investigations will facilitate the development of superior apple varieties with augmented fruit quality and resilience in the context of climate change challenges.

## 3. Conclusions

The ongoing climate change leads to adverse environmental conditions and poses a major threat to agriculture, impacting crop yield and quality. Tolerance to abiotic stresses and resistance to biotic stresses of economically important crops is consequently significantly affected. Crop improvement and breeding for novel varieties with enhanced yield, quality, and stress resilience have become imperative to face current and future climate challenges and ensure food security for an ever-growing human population. In recent decades, emphasis has been given to the exploitation of the rich genetic diversity of indigenous varieties and landraces of agronomically important crops. The rapid advancements in genomic technologies have paved the way for characterizing this highly valuable germplasm at the molecular level and discovering significant genes and gene networks associated with the establishment of advantageous traits that could lead to superior genotypes and climate-resilient crops.

To our knowledge, direct commercial release of improved germplasm derived from indigenous varieties and landraces using novel genomic tools has not yet been reported for the species discussed in this paper. Herein, we provided an overview of the research efforts and progress achieved in the employment of advanced genomic tools to mine the vast genetic variability of landraces and indigenous varieties of important crops cultivated in the Mediterranean region. Integrating the valuable outcomes from the development of pangenome references, novel genotyping platforms, multi-omics approaches, and genome-wide association studies (GWAS) linking crucial genes and genomic regions with important agronomical characteristics has provided a wealth of information for elucidating the genetic architecture of key traits. As new information rapidly emerges through the technologies described, it is anticipated that introgression lines incorporating advantageous traits from indigenous varieties and landraces on elite background, especially for melon and tomato, will be developed and released soon and improved crops will be feasible through modern breeding.

## Figures and Tables

**Table 1:** Landraces with resistance or susceptibility to various pests and diseases that used as parents for RIL populations.

Landraces	Type	Indication	Disease/Pest	References
ZN016	Yard-long bean	Resistant	Rust	Wu et al. [62]
			Powdery mildew	Wu et al. [66]
Donsin, Moussa	Cowpea	Resistant	Brown blotch	Thio et al. [67]
Gorom (SuVita 2)	Cowpea	Resistant	*Macrophomina phaseolina*	Huynh et al. [68]
			*Striga gesnerioides*	
Keffi local	Cowpea	Susceptible	Aphids	Omoigui et al. [69]

**Table 2:** Number of genes/loci identified to be associated with biotic stress resistance in cowpea landraces and type of molecular markers used.

Biotic Stresses	Molecular Marker	Number of Candidate Genes/Loci	References
Weevil	SNPs	11	Kpoviessi et al. [94]
Aphids	SNPs	3	Ongom et al. [95]
Fusarium wilt	SNPs	30	Dong et al. [96]
Cowpea Mosaic Virus (CPMV)	SNPs	-	Bhattarai et al. [97]

## Data Availability

Not applicable.
